# Ultrasound-Guided Placement of Tunneled Hemodialysis Catheters Using Direct Atrial Visualization: Clinical and Functional Results at One Year Follow-Up

**DOI:** 10.3390/medicina62071236

**Published:** 2026-06-26

**Authors:** Jesús E. García-Solano, Antoni N. Osorio Mendoza, José C. De La Flor, Avinash Chandu Nanwani, Carlos Narváez-Mejia, Juan Lluncor-Vasquez, Esperanza Moral Berrio, Celia Rodríguez Tudero, Carlos Cruzado-Pizarro, Elena Jiménez Mayor, Michael Cieza Terrones, Bernardo Moguel-Gonzáles

**Affiliations:** 1 Department of Nephrology, Hospital Cayetano Heredia, Lima 15002, Peru; jesus.garcia.s@upch.pe (J.E.G.-S.); antoni.osorio@upch.pe (A.N.O.M.); 2 Department of Nephrology, Hospital Central de la Defensa Gomez Ulla, 28047 Madrid, Spain; 3 Department of Medicine and Medical Specialties, Faculty of Medicine, Alcala University, 28805 Madrid, Spain; 4 Department of Nephrology, Hospital General de Fuerteventura, 35600 Fuerteventura, Spain; achanan@gobiernodecanarias.org; 5 Department of Nephrology, Hospital Universitario Puerta del Mar, 11009 Cádiz, Spain; ceduardo.narvaez.sspa@junta-deandalucia.es; 6 Department of Nephrology, Hospital Dos de Mayo, Lima 15003, Peru; juan.lluncor@upch.pe; 7 Department of Nephrology, Hospital General Universitario de Ciudad Real, 13005 Ciudad Real, Spain; emoral@sescam.jccm.es; 8 Department of Nephrology, Hospital Universitario de Salamanca, 37007 Salamanca, Spain; crodrigueztudero@usal.es; 9 Surgery Department, Faculty of Medicine, University of Salamanca, 37007 Salamanca, Spain; 10 Department of Nephrology, Hospital Nacional Ramiro Prialé, Huancayo 12006, Peru; ccruzado8@gmail.com; 11 Department of Nephrology, Hospital Santa Bárbara, 42003 Soria, Spain; ejimenezmay@saludcastillayleon.es; 12 Department of Engineering, Faculty of Science and Engineering, Peruana Cayetano Heredia University, Lima 15012, Peru; michael.cieza@upch.pe; 13 Interventional Nephrology Unit, Ignacio Chávez National Institute of Cardiology, Mexico City 14080, Mexico; nefrointervencioninc@gmail.com

**Keywords:** hemodialysis, tunneled central venous catheter, ultrasound, interventional nephrology, right atrium

## Abstract

*Background and Objectives*: Ultrasound-guided placement of tunneled hemodialysis catheters may be useful when fluoroscopy is unavailable or radiation exposure should be avoided. This study describes a technique based on direct ultrasound visualization of the metallic guidewire within the right atrium and evaluates one-year clinical and functional outcomes. *Materials and Methods*: We conducted a single-center retrospective observational study of 319 adult hemodialysis patients undergoing tunneled catheter placement. The technique combined intravascular guidewire length measurement with subcostal ultrasound visualization of the guidewire tip in the right atrium. Baseline characteristics, insertion site, immediate complications, blood flow rate, Kt/V, and extracorporeal circuit pressures were analyzed. *Results*: Mean age was 55.9 ± 14.5 years, and 58.6% were women. The main causes of chronic kidney disease were diabetes mellitus and arterial hypertension. Mean blood flow rate was 349 mL/min at 3 months and 357 mL/min at 12 months. Mean Kt/V at 12 months was 1.57. No catheter malpositions requiring immediate repositioning were documented. Procedure-related complications were infrequent and mainly local. *Conclusions*: Ultrasound-guided tunneled catheter placement using direct visualization of the guidewire within the right atrium was technically feasible and associated with favorable functional parameters and few immediate complications. Given the retrospective design and lack of a comparative group, these findings should be interpreted with caution. Prospective comparative studies are needed to confirm safety, reproducibility, and clinical utility.

## 1. Introduction

The prevalence of chronic kidney disease (CKD) has increased considerably worldwide in recent years. In Peru, the prevalence increased over the last decade to 648 patients per million population, while hemodialysis became the most frequently used renal replacement therapy modality, with a rate of 363 patients per million population; only 11% of patients have achieved kidney transplantation [1,2,3].

By 2024, approximately 80% of patients receiving renal replacement therapy (RRT) were treated with hemodialysis, which requires adequate vascular access. Although the arteriovenous fistula (AVF) remains the ideal access because of its better clinical outcomes compared with hemodialysis catheters, in our setting its creation is often delayed, and a substantial proportion of patients do not have adequate blood vessels for its creation by cardiovascular surgery [1,4].

In our setting, most patients initiate dialysis urgently and depend on non-tunneled hemodialysis catheters because of their greater availability and lower cost. Therefore, in patients who require tunneled hemodialysis catheters, it is essential to ensure adequate functionality while minimizing complications and catheter dysfunction [3,5].

Correct placement of tunneled hemodialysis central venous catheters (tHDCVCs) is fundamental to ensure adequate blood flow (QB), reduce complications, and prolong vascular access survival. The Kidney Disease Outcomes Quality Initiative (KDOQI) 2019 guidelines recommend that the catheter tip be located in the right atrium or at the cavoatrial junction, with the aim of optimizing its function and reducing cardiovascular risks [6]. Fluoroscopy has been the reference method for confirming catheter position; however, it involves radiation exposure, specific infrastructure, higher costs, and longer procedure times [7,8].

In recent years, ultrasound-guided alternatives have been developed with success and safety rates comparable to those obtained with fluoroscopy [7,9,10]. In particular, direct ultrasonographic visualization of the guidewire in the right atrium through a subcostal view, combined with intravascular length measurement, has emerged as a useful strategy to reduce catheter malposition [7,10].

Recently, an interventional standard for tHDCVC placement has been proposed, highlighting the role of ultrasound and alternative methods to fluoroscopy for precise catheter tip localization, as well as the importance of operator experience and structured interventional nephrology programs. However, clinical evidence regarding the functional performance and medium-term stability of these techniques in large cohorts and real-world clinical practice settings remains limited [11].

Similarly, the Spanish Clinical Guideline for Vascular Access highlights the importance of a multidisciplinary approach, venous capital preservation, and the systematic adoption of ultrasound-guided techniques to improve the safety and efficacy of vascular access in hemodialysis [12]. From the perspective of diagnostic and interventional nephrology, the use of real-time ultrasound allows CVC placement to become a bedside, radiation-free, reproducible, and resource-efficient procedure, aligned with the expansion of the Point-of-Care Ultrasound (POCUS) model and contemporary interventional nephrology [13,14,15]. In addition, the increasing availability of portable ultrasound devices and the evidence supporting their ability to improve success rates, reduce complications, shorten procedure times, and optimize hospital costs reinforce the need to standardize these techniques [5,8].

In this context, the present study proposes a new practical, safe, and reproducible technique for the placement of high-flow hemodialysis CVCs, based on direct ultrasonographic visualization of the guidewire tip within the right atrium and on intravascular length measurement as a reference to determining the optimal catheter insertion length. This strategy aims to minimize malposition, optimize dialysis performance, and reduce reliance on fluoroscopy, providing clinical evidence from a large patient cohort and contributing to the practical validation of one of the approaches described within the current interventional standard [11].

## 2. Materials and Methods

### 2.1. Trial Design and Participants

This is a single-center, retrospective observational study. We included 319 patients older than 18 years who were receiving RRT with hemodialysis; the study was conducted at the Interventional Nephrology Unit of Hospital Cayetano Heredia in Lima, Peru. All data were collected by expert nephrologists in a database specifically designed for this purpose, retrieving all available information from medical and departmental history.

During the study period, hemodialysis was the predominant RRT modality, reflecting the local organization of care, resource availability, and the clinical characteristics of the population served. Peritoneal dialysis was also available as a therapeutic alternative; however, approximately 20 patients were enrolled in this program during the same period, representing a relatively smaller proportion of the total population receiving RRT at our center.

In the absence of an established advanced chronic kidney disease (ACKD) program, most patients had their first contact with nephrology in the setting of an urgent event requiring dialysis initiation, leading to the use of a non-tunneled catheter as the initial vascular access.

At our institution, temporary hemodialysis catheters are placed by nephrologists trained in vascular access procedures and ultrasound-guided central venous catheterization. These procedures are not performed by anesthesiologists or vascular surgeons at our center.

Notably, approximately 50% of patients did undergo evaluation by cardiovascular surgery; however, they were deemed unsuitable for AVF creation. Before catheter placement, signed informed consent was required both for the procedure and for inclusion in the study. In addition, the necessary data were collected, including comorbidities and other general characteristics; patients were also asked about the reasons for not having an arteriovenous fistula created. No patients with a known diagnosis of thrombophilia were identified in the cohort; therefore, this variable was not included in the final analysis. No patients were receiving active oral anticoagulant therapy at the time of the procedure. In patients previously treated with oral anticoagulants, these medications were discontinued 3 to 5 days before catheter placement according to institutional protocol and individualized perioperative risk assessment. After tunneled catheter placement, patients underwent continuous quarterly follow-up for one year to collect variables related to catheter function, including QB, arterial and venous system pressures, as well as dialysis adequacy parameters (Kt/V).

**Figure 1:**
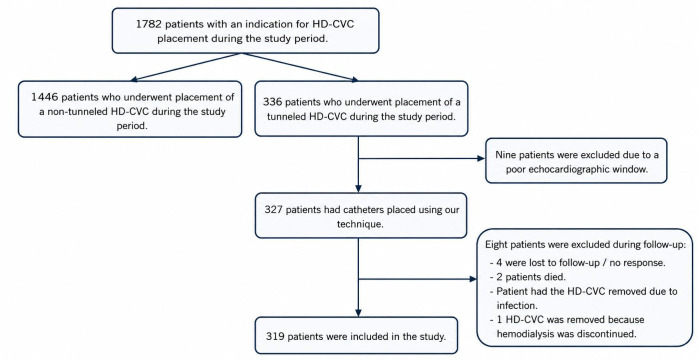
Flowchart of patient inclusion and exclusion criteria. tHDCVC: tunneled hemodialysis central venous catheter.

The only exclusion criterion was the presence of an inadequate ultrasound window that prevented proper visualization of the cardiovascular structures (Figure 1). All patients signed informed consent before the procedure. All procedures included in the study were performed under the direct supervision and active participation of the same interventional nephrologist, who had experience in tunneled catheter implantation and ultrasound-guided procedures. The involvement of the same primary operator throughout the study period allowed the maintenance of a homogeneous technique and reduced variability related to differences in technical experience.

In addition, the procedures were performed with the assistance of medical residents who had been previously trained in the technique under direct supervision. A total of 8 residents participated during the study period, 4 residents per year, all of whom underwent progressive supervised training before actively participating in the procedures.

Access site selection followed a hierarchical approach: right internal jugular vein as first choice, left internal jugular vein in cases of vascular exhaustion or anatomical limitations, and femoral veins as the last option. The technique used is aligned with the principles of the recently proposed interventional nephrology standard, as it prioritizes direct anatomical visualization, procedural safety, and standardization of the approach [11].

Of the 319 patients, 289 underwent exchange of a temporary catheter for a tunneled catheter using an over-the-wire technique, after radiographic verification of the appropriate venous position of the temporary catheter. In the 30 de novo placement cases, ultrasound guidance was used to identify and puncture the target vein before guidewire and catheter advancement.

As a quality control measure, data were extracted from institutional records and subsequently verified through cross-checking with the hospital database. An assessment of data consistency and completeness was performed before statistical analysis, and missing data were retrieved when available from the original records.

The primary objective of this study was to assess the performance of tunneled hemodialysis central venous catheters inserted using the novel technique and to document associated procedural complications.

### 2.2. Description of the Ultrasound-Guided Tunneled Hemodialysis Central Venous Catheter Placement Procedure

**Figure 2:**
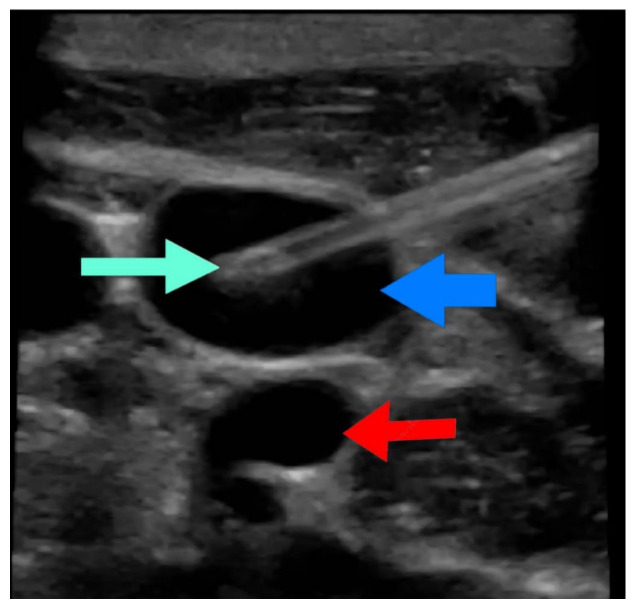
Cross-section view of the internal jugular vein (blue arrow), carotid artery (red arrow), and trocar needle (green arrow).

Using a linear ultrasound transducer for venous access selection, the right internal jugular vein was the first choice, as it provides a straighter course to the right atrium and a lower risk of stenosis or venous collapse. For this access, 14.5 Fr × 28 cm double-lumen polyurethane catheters with a split-tip configuration, Arrow^®^ brand (Chicago, IL, USA), were used (Figure 2).

In cases where this approach was not feasible, either due to vascular exhaustion or other limitations, the left internal jugular vein was used. For this access, a 14.5 Fr × 33 cm double-lumen polyurethane catheter with a step-tip configuration, Mediplus^®^ brand (High Wycombe, UK), was employed. This configuration was used exclusively because of the longer anatomical course; the use of split-tip catheters may be associated with kinking or entrapment, which can make insertion difficult.

As a last option, the femoral veins were used. For this access, a 14.5 Fr × 55 cm double-lumen polyurethane catheter was employed, also with a step-tip configuration, Mediplus^®^ brand. Its greater length allowed visualization of the guidewire within the atrial cavity, as well as the catheter tip.

Once the best access for the patient had been selected, and after informed consent had been signed both for catheter placement and for participation in the study, strict aseptic and antiseptic measures were carried out. These included surgical aseptic technique with surgical hand disinfection, sterile gloves and gown, facemask, and a minimum sterile surgical field. Skin antisepsis was performed using a two-component solution consisting of alcohol and a residual-acting agent (chlorhexidine or octenidine dihydrochloride). Subsequently, all patients received local anesthesia with 2% lidocaine without epinephrine under continuous ultrasound guidance; no patient required sedation.

**Figure 3:**
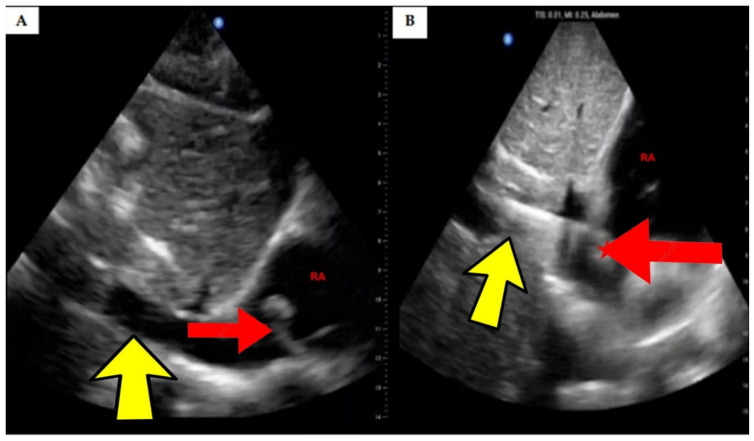
Guidewire (red arrow) visualized in the right atrium (RA) via the superior vena cava (**A**) and the inferior vena cava (yellow arrow) (**B**) using the xiphoid window.

Venous puncture was performed in real-time, confirming the entry of the needle into the venous lumen using both transverse and longitudinal views. After confirming the correct needle position, a J-tip guidewire was introduced, and the needle was withdrawn. Subsequently, using a subcostal echocardiographic window, the guidewire was advanced until the J-tip was clearly and directly visualized within the right atrium, always carefully avoiding contact with the tricuspid valve (Figure 3A,B). Unlike the standard technique, the guidewire was deliberately advanced into the right heart chambers while obtaining a subcostal four-chamber view, allowing direct visualization of the J-tip within the right atrium (Figure 3B). This direct anatomical confirmation eliminated the need for anthropometric calculations or estimation based on external landmarks, whose accuracy is known to vary according to patient body habitus, sex, sternal distance, and thoracic depth. Although intracavitary electrocardiographic monitoring is the reference standard, the literature supports that conventional continuous cardiac monitoring allows timely detection of rhythm disturbances during guidewire advancement and contributes to procedural safety. Therefore, a multiparameter monitor with real-time electrocardiographic (ECG) visualization was used to detect potential procedure-related cardiac arrhythmias during metallic guidewire advancement [5,7,16].

**Figure 4:**
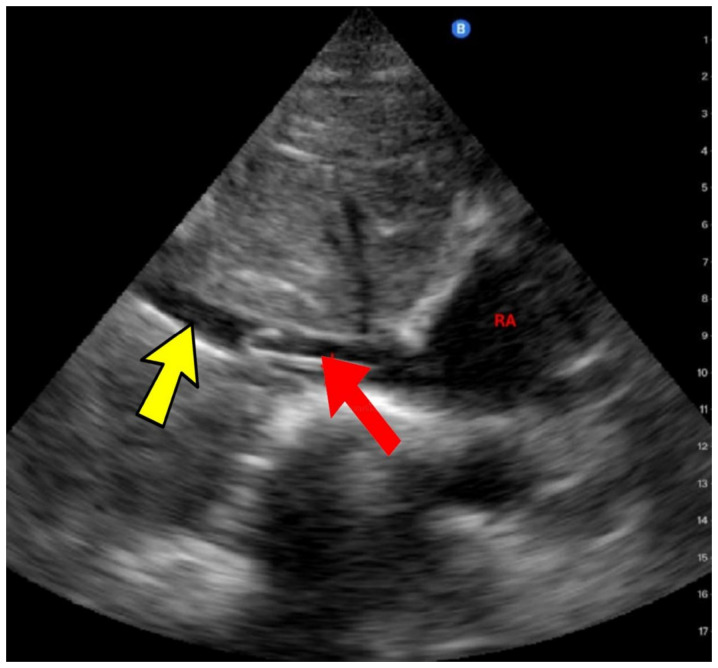
Guidewire (red arrow) visualized in the inferior vena cava (yellow arrow) via the internal jugular vein using the xiphoid window. RA: Right atrium.

**Figure 5:**
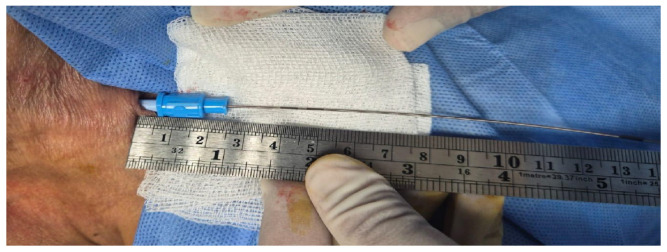
External measurement of the marked guidewire.

Once the guidewire tip was observed in the right atrium, the skin entry point was marked, and the guidewire was progressively withdrawn until the tip was positioned again in the atrium (Figure 4). The recorded intravascular length corresponded to the actual distance between the insertion point and the right atrium, enabling the determination of the exact catheter length to be inserted (Figure 4 and Figure 5). Recent publications highlight that anatomical variability makes fixed catheter lengths a suboptimal method, whereas directly measuring the guidewire allows for precise individualization of the length to be introduced [7,8,11].

In Figure 4, the metallic guidewire tip is visualized within the inferior vena cava during ultrasound-guided navigation. Continuous real-time ultrasound guidance allowed controlled withdrawal of the guidewire by approximately 2 cm, achieving final positioning of the guidewire tip within the right atrium.

### 2.3. Intravascular Measurement and Tunneling

Once the guidewire tip was confirmed to be in the right atrium, the skin entry point was fixed, and the guidewire was slowly withdrawn until its end was positioned at the desired location. The inserted intravascular length was recorded using the external marks on the guidewire (Figure 5). This measurement served as a reference to determine the optimal catheter insertion length.

**Figure 6:**
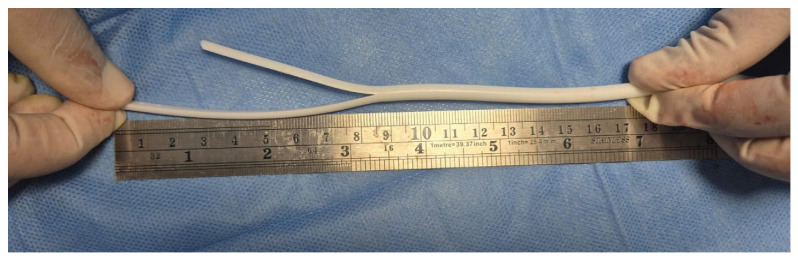
High-flow catheter measurement based on marked guidewire length.

Subsequently, tract dilation and subcutaneous tunneling were performed. In female patients, tunneling was carried out using a curved trajectory to avoid proximity to the mammary gland, thereby reducing discomfort and minimizing the risk of mastitis in the event of future tunnel infection. Finally, the high-flow catheter was inserted according to the previously measured length (Figure 6).

### 2.4. Confirmation of Catheter Position

**Figure 7:**
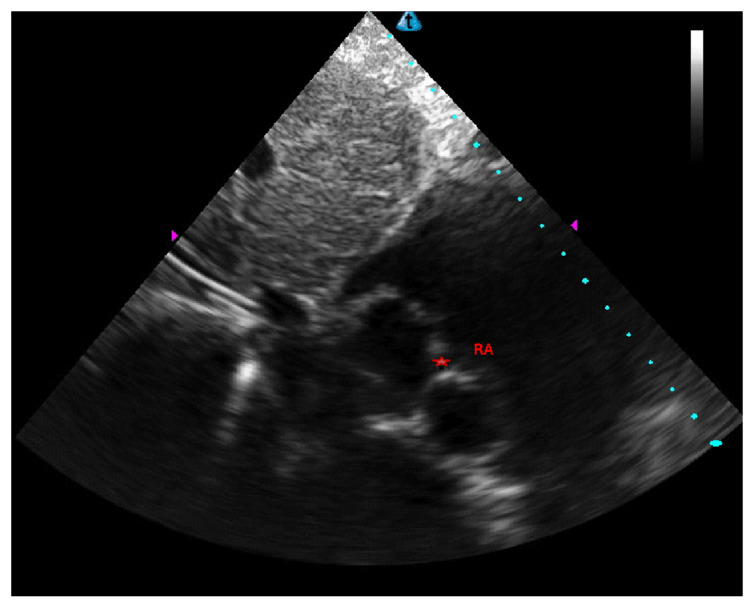
High-flow catheter tip (red star) visualized in the right atrium (RA).

The correct position of the catheter tip was verified using two ultrasound methods: (1) Direct visualization of the catheter tip within the right atrium using a subcostal window. (2) The bubble test, which consists of injecting agitated saline, with microbubbles observed immediately entering the right atrium (Figure 7), indicating correct catheter tip placement [7,10]. For femoral accesses, we used a longer catheter (14.5 Fr × 55 cm); it should be noted that femoral access was used only in patients in whom the jugular veins could not be accessed because of vascular exhaustion, with the tip observed to be within the indicated parameters, in addition to performing the bubble test.

All patients received a postprocedural chest X-ray to document the position of the catheter and exclude complications [7,8].

**Figure 8:**
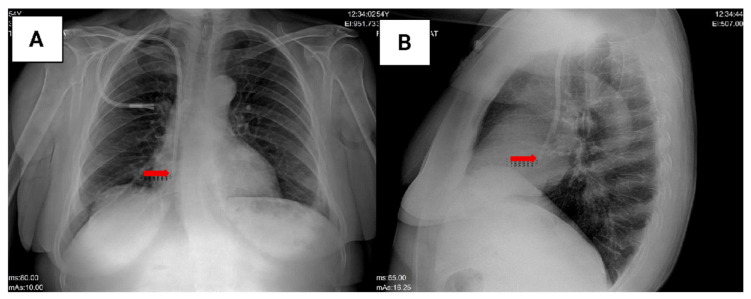
(**A**,**B**) Post-procedural chest radiograph following tHDCVCs placement. Catheter tip indicated by red arrow.

As can be seen, correct placement of a catheter in the right internal jugular vein is demonstrated, with the tip in the atrium, both on the frontal view (Figure 8A) and the lateral view (Figure 8B). In this case, we can observe the curved tunnel being placed in a female patient, keeping it away from the mammary gland.

**Figure 9:**
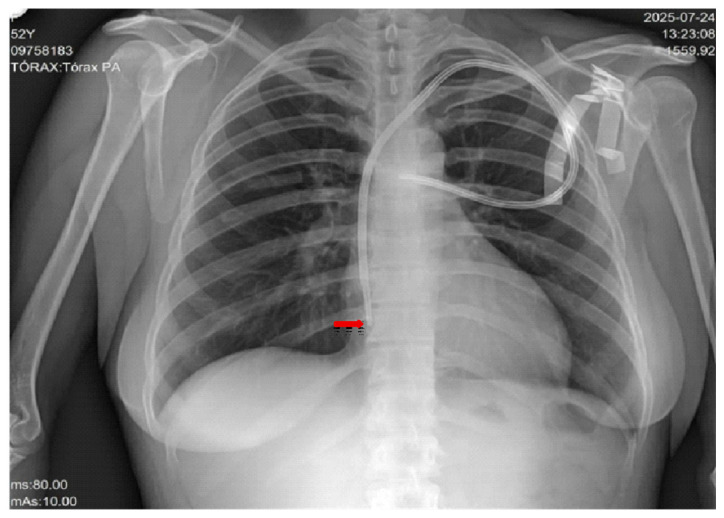
Post-procedural chest X-ray showing catheter placement via the left internal jugular vein in a female patient. Catheter tip indicated by red arrow.

In Figure 9, we can observe the correct placement in the left internal jugular vein, also in a female patient, with a curved tunnel. As we previously mentioned, for the left internal jugular vein, because the distance from the insertion point to the right atrium is greater, a longer catheter is used.

**Figure 10:**
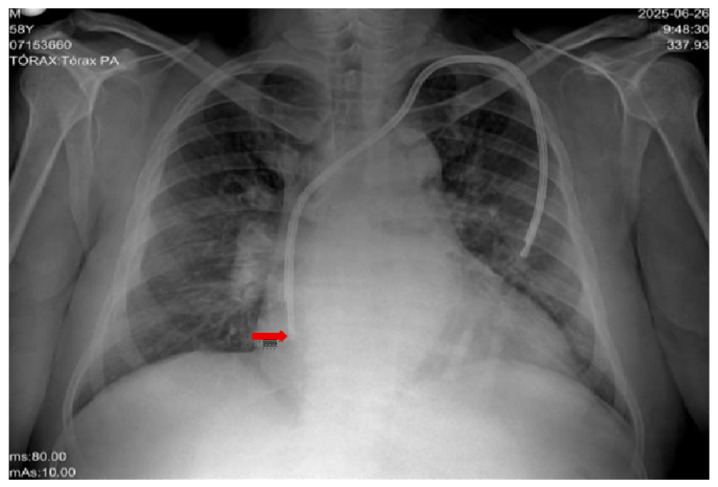
Post-procedural radiographic image after catheter placement in the left internal jugular vein in a male patient. Catheter tip indicated by red arrow.

In Figure 10, also in the left internal jugular vein but in a male patient, note the straight tunnel.

**Figure 11:**
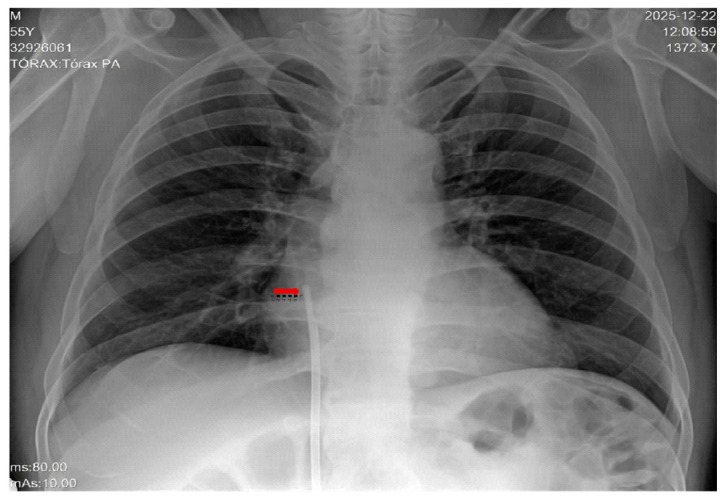
Post-procedural radiograph showing catheter placement via the right femoral vein. Catheter tip indicated by red arrow.

In Figure 11, we can see a radiograph obtained after placement of a catheter through femoral access; note that, because a longer catheter was used, its tip reaches the atrial cavity.

All patients were monitored for two hours to detect early complications such as arrhythmia, hematomas, or bleeding from the subcutaneous tunnel. Likewise, after radiological confirmation, there was no need for catheter repositioning.

### 2.5. Statistical Analysis

Continuous variables were presented as means and standard deviations (SD) or medians and interquartile ranges (IQR), depending on data distribution. Categorical variables were expressed as frequencies and valid percentages. Comparisons between the same subjects at different time points were performed using the paired Student’s *t*-test. All quarterly follow-up data were complete, and no missing values were recorded during the study period. Statistical analyses were performed using STATA 14.0 (Stata Statistical Software: Release 14. College Station, TX, USA: StataCorp LP).

## 3. Results

A total of 319 patients who received a tHDCVC were included. The mean age was 55.9 ± 14.5 years, and 187 (58.6%) were women. The predominant etiologies of CKD were type 2 diabetes mellitus (T2DM) (45.7%), arterial hypertension (AHT) (30.6%), and autosomal dominant polycystic kidney disease (ADPKD) (7%). Transient premature ventricular contractions were observed in 4 patients; these occurred during advancement of the metallic guidewire and resolved after withdrawing it by approximately 1 cm. No sustained arrhythmias or clinically significant events were recorded (Table 1).

Immediate complications were defined as those occurring during the procedure or within the first 2 h after the procedure. Immediate procedure-related complications occurred in 11 of 319 patients (3.4%), with each event occurring in a different patient. The complications included hematoma, arterial puncture, bleeding, and a single case of hemothorax (Table 1).

The hemothorax occurred after femoral vein access, was diagnosed by post-procedural chest radiography, and was managed conservatively with a favorable outcome (Table 1).

The analysis of effective BFR (Blood Flow Rate) showed consistently elevated values during follow-up. The mean BFR at 3 months was 349 mL/min, while at 12 months it increased slightly to 357 mL/min, remaining within the recommended ranges for adequate dialysis efficiency in both periods [17].

All patients received intermittent hemodialysis, with session durations between 3.5 and 4 h. Low-flux dialyzers were used, according to the dialysis center’s policy. Although some guidelines recommend BFR above 400 mL/min, per the dialysis centers’ practice, the patients were maintained at BFR ≥ 350 mL/min for better intradialytic tolerability (Table 2). This did not compromise adequate dialysis dosing, as confirmed by their Kt/V measurements [17].

The mean Kt/V at 12 months was 1.5, with a range between 1.3 and 1.6, meeting international standards for dialysis adequacy (≥1.4) [6]. The Kt/V at 3 months, available only in patients with complete data, also remained within values considered acceptable (Table 2).

The arterial and venous pressures recorded at both 3 and 12 months remained within the expected physiological ranges for functional tunneled catheters. No excessively negative or elevated pressures were observed that would suggest tip malposition, significant stenosis, or hemodynamic alterations in the extracorporeal circuit.

All 319 patients completed follow-up at 3, 6, 9, and 12 months for all study variables. No missing data were recorded; therefore, no imputation or sensitivity analyses for missing data were performed.

No catheter malpositions were documented, nor was immediate repositioning required after insertion. Documented complications were few and primarily local, including hematomas and mild bleeding in the tunnel tract or insertion site. No cases of procedure-related pneumothorax were recorded in our cohort. Only one patient developed a hemothorax. Given that pneumothorax is a recognized mechanical complication of central venous catheter placement for hemodialysis, the absence of this event may be related, at least in part, to the systematic use of real-time ultrasound guidance for vascular identification, puncture-site selection, and venous cannulation.

In addition, the reasons why patients did not have a created arteriovenous fistula at the time of catheter placement were analyzed (Table 3).

In the group of other reasons (24.4%), heterogeneous causes with low individual frequency were included, among them specific comorbidities (stroke, severe anemia, oncologic disease) and functional limitations (use of a wheelchair or crutches).

All patients completed a 12-month follow-up. During this period only 5 patients (1.6%) had catheter dysfunction that required an intervention to maintain patency. Additionally, 18 patients experienced at least one infectious episode.

## 4. Discussion

The present study describes a single-center experience with ultrasound-guided placement of tHDCVCs based on direct visualization of the guidewire tip in the right atrium and intravascular length measurement. Our findings suggest that this approach is technically feasible and was associated with favorable functional outcomes and a low rate of immediate procedure-related complications. These findings are consistent with the principles of the recently proposed interventional standard for tunneled CVC placement, which emphasizes ultrasound-based strategies, operator expertise, and standardized procedural workflows [11].

One of the most relevant findings of this study is the medium-term functional stability of vascular access, evidenced by blood flow rate (BFR) values consistently exceeding minimum recommendations and an adequate mean Kt/V at 12 months. This performance is likely related to correct anatomical positioning of the catheter tip in the right atrium or at the cavoatrial junction, which promotes efficient flow dynamics and minimizes recirculation. The absence of malposition and the limited need for corrective maneuvers reinforce the precision and reliability of the ultrasound-guided method employed.

Several previous studies have demonstrated that ultrasound can safely replace fluoroscopy in the placement of tunneled CVCs when standardized techniques are applied and procedures are performed by experienced operators [6,7,9]. In this context, the recently described interventional standard reinforces this approach and highlights the need for clinical evidence evaluating not only technical feasibility but also the functional performance of vascular access in the medium term [11], an aspect directly addressed in the present study. Our results are consistent with this evidence and provide added value by demonstrating, in a large cohort within a real-world clinical practice setting, that direct intracardiac anatomical confirmation reduces dependence on anthropometric calculations [18].

The observed BFR values (349–357 mL/min) substantially exceed the minimums suggested by KDOQI guidelines and reflect adequate hemodynamic performance of the catheters [17]. Likewise, the mean Kt/V close to 1.6 reinforces the relationship between optimal catheter tip positioning and adequate dialysis efficiency, supporting the hypothesis that precise anatomical control favors access function and reduces recirculation.

From the perspective of diagnostic and interventional nephrology, direct ultrasound visualization of the guidewire and catheter in the right atrium transforms a procedure traditionally based on anatomical estimations into an intervention guided by real-time anatomy. This approach increases technical precision and procedural safety by allowing the operator to identify and correct inappropriate trajectories during guidewire advancement, avoid excessive intracardiac manipulation, and reduce the risk of immediate complications. In our study, guidewire advancement into the right atrium was performed under direct ultrasound visualization and continuous electrocardiographic monitoring. Only transient extrasystoles were observed in 4 patients, resolving after partial withdrawal of the guidewire, without sustained arrhythmias or other clinically significant cardiac complications. These findings support the safety of the procedure when performed under appropriate monitoring. Nevertheless, recent technologies, such as newer intracavitary ECG systems and nitinol mini-guidewires, aim to further reduce the need for extensive intracardiac guidewire advancement. In this regard, Cirigliano et al. reported the non-inferiority of newer intracavitary ECG-based approaches, representing a relevant evolution in catheter tip localization strategies. This highlights that our technique should not be interpreted as competing with contemporary tip-location methods, but rather as an ultrasound-based strategy that may complement them, particularly in settings where direct anatomical confirmation is feasible [19].

Our protocol shares principles with contemporary approaches for ultrasound-guided catheter tip navigation and localization, including the ECHOTIP protocol and its subsequent updates, the bubble test, intracavitary ECG, and post-procedural radiographic assessment. However, its main distinguishing feature is the direct ultrasound visualization of the metallic guidewire tip within the right atrium, followed by individualized measurement of the distance to the insertion site. This strategy allows a more accurate determination of catheter insertion length and may be particularly useful in tunneled hemodialysis catheter placement, where catheter caliber, tunneling trajectory, and final tip position are critical for both safety and long-term function [20,21].

In addition, the ECHOTIP protocol and its subsequent updates are not restricted to non-tunneled central venous catheters but have also been applied to tunneled catheters and totally implantable venous access devices. Therefore, our technique should be interpreted as a complementary variant within the current framework of ultrasound-based catheter tip navigation and localization.

Finally, it is important to emphasize that intracardiac contact during the initial navigation phase is primarily related to the metallic guidewire rather than to the final caliber of the implanted catheter. In our experience, the diameter of the guidewire is comparable regardless of whether it is used for the placement of large-bore tunneled hemodialysis catheters or smaller-caliber, low-flow central venous catheters. Therefore, the purpose of our approach was not to increase intracardiac manipulation, but rather to perform this critical step under direct ultrasound visualization and continuous electrocardiographic monitoring. This strategy was intended to enhance procedural control, allow early recognition and correction of inappropriate guidewire positioning, and optimize final catheter tip localization.

The low complication rate observed in our series, mainly limited to minor local events, is consistent with findings described in the literature [5,8], and suggests that the ultrasound-guided approach may contribute to reducing morbidity associated with tunneled CVC placement. Although ultrasound guidance does not eliminate the risk of infection or late catheter dysfunction, correct initial tip positioning could favorably influence access durability and reduce the need for subsequent manipulations.

Another relevant aspect of this study is its applicability in clinical settings with limited fluoroscopy availability or in scenarios where reducing radiation exposure—for both patients and healthcare personnel—is prioritized. The ability to perform the procedure at the bedside using widely available ultrasound equipment reinforces the role of interventional nephrology as a discipline oriented toward safe, efficient, and cost-effective procedures, consistent with the expansion of the point-of-care ultrasound (POCUS) model.

### 4.1. Limitations

This study has several limitations. Its retrospective, single-center design and the absence of a control group (fluoroscopy, intracavitary ECG, or ECHOTIP-guided catheter placement) limit direct comparative conclusions. In addition, the quality of the subcostal ultrasound window may be suboptimal in certain patients, potentially requiring the use of complementary confirmation methods. The technique was performed by a single experienced operator, which may affect the generalizability and reproducibility of the results in other clinical settings. Future multicenter prospective studies including comparator groups are needed to validate these findings.

### 4.2. Vascular Access Considerations

In the analysis of recorded reasons for non-creation of an arteriovenous fistula, we observed that one in three patients had anatomical or clinical limitations preventing the creation of autologous vascular access following cardiovascular surgery evaluation, which constitutes a relevant factor in the persistent use of catheters.

The second most frequent cause was that patients were still awaiting evaluation, highlighting the existence of a group potentially eligible for definitive vascular access creation. This percentage may represent an opportunity for improvement within vascular access programs through optimization of evaluation processes and timely referral.

Additionally, 11.6% of patients had a history of failed, thrombosed, or nonfunctioning AVF, reflecting the well-documented difficulties associated with vascular access maturation and maintenance in patients with advanced chronic kidney disease.

Among other causes, advanced age was identified—a condition often associated with greater vascular fragility, multiple comorbidities, and limited life expectancy. A smaller proportion of patients declined AVF creation, underscoring the importance of patient education and shared decision-making in vascular access planning.

In patients with limited or exhausted vascular access options for hemodialysis, peritoneal dialysis may be considered as an alternative kidney replacement therapy modality. However, this decision must be individualized and depends on abdominal conditions, functional status, self-care capacity, family support, patient preferences, and the availability of specialized institutional follow-up.

### 4.3. Advantages of Ultrasound-Based Visualization

Ultrasound-based visualization of catheter tip position offers advantages over fluoroscopy, as it allows better delineation of anatomical structures without the need for contrast agents. Complementary imaging methods broaden the range of available techniques and, consequently, improve access to catheter implantation procedures.

## 5. Conclusions

In this single-center retrospective cohort, ultrasound-guided placement of tHDCVCs using direct visualization of the guidewire tip in the right atrium and intravascular length measurement was technically feasible and was associated with favorable functional access performance, stable blood flow rates, satisfactory dialysis adequacy, and a low rate of immediate procedure-related complications. No catheter malposition requiring immediate repositioning was documented in our cohort.

These findings suggest that real-time ultrasound guidance may be a useful adjunct for optimizing tunneled hemodialysis catheter placement, particularly in settings where fluoroscopy is not readily available or where radiation-free bedside procedures are preferred. However, given the retrospective design, single-center setting, and absence of a comparator group, this technique should be interpreted as a complementary strategy rather than a replacement for established fluoroscopy-, intracavitary ECG-, or structured ultrasound-based tip localization methods. Prospective multicenter comparative studies are needed to further evaluate its safety, reproducibility, learning curve, and potential clinical advantages.

## Figures and Tables

**Table 1:** Baseline demographic data.

*n* = 319	
	**Demographics**
55.9 (14.5)	Age—years, mean (SD)
187 (58.6)	Sex—female, *n* (%)
	**Etiology of CKD, ** ***n*** **(%)**
145 (45.7)	Diabetes Mellitus
97 (30.6)	Arterial hypertension
22 (7)	Autosomal dominant polycystic kidney disease (ADPKD)
11 (3.5)	Primary glomerulonephritis
11 (3.5)	Renal hypoplasia
33 (10.1)	Other
	**Insertion Site, ** ***n*** **(%)**
182/52 (57.1/16.3)	Internal jugular vein (Right/Left)
56/29 (17.6/9.1)	Femoral vein (Right/Left)
	**Number of Punctures, ** ***n*** **(%)**
306 (95.9)	1
11 (3.4)	2
1 (0.3)	3
1 (0.3)	4
	**Tunnel Type, ** ***n*** **(%)**
143 (44.8)	Straight
176 (55.2)	Curved
	**Immediate Complications**
4 (1.25)	Hematoma
3 (0.94)	Arterial puncture
3 (0.94)	Bleeding
1 (0.3)	Hemothorax

Data are shown as mean (SD) or number (percentage). Percentages calculated based on total complications (*n* = 11). CKD: Chronic kidney disease. SD: Standard deviation.

**Table 2:** Functional catheter parameters during follow-up.

Variable	Time	Min–Max	Mean ± SD	*p*-Value *
Kt/V	3 months (n319)	1.1–1.6	1.4 ± 0.1	
	12 months (n319)	1.3–1.6	1.5 ± 0.1	<0.001
DAP, mmHg	3 months (n319)	−249.0–−80.0	−167.8 ± 51.0	
	12 months (n319)	−249.0–−80.0	−161.1 ± 51.6	0.11
DVP, mmHg	3 months (n319)	81.0–199.0	141.9 ± 33.5	
	12 months (n319)	80.0–199.0	138.2 ± 33.7	0.17
BFR, mL/min	3 months (n319)	300.0–400.0	349.1 ± 9.7	
	6 months (n319)	300.0–400.0	355.8 ± 19.2	<0.001
	9 months (n319)	300.0–400.0	356.4 ± 18.5	<0.001
	12 months (n319)	300.0–400.0	357.2 ± 19.3	<0.001

Data are shown as mean (SD, standard deviation), DAP: dialysis arterial pressure, BFR: Blood Flow Rate, DVP: dialysis venous pressure, Min: minimum, Max: maximum. * *p*-value vs. 3 months.

**Table 3:** Reasons why patients did not have a created arteriovenous fistula.

Cause of Non-Creation of Arteriovenous Fistula	%
Unfavorable vascular conditions	31.6
Awaiting evaluation	15.1
Failed Arteriovenous Fistula	11.6
Advanced age	9.3
Patient does not want	4.4
Unfavorable cardiac conditions	3.6
Other reasons	24.4

## Data Availability

No new data were created or analyzed in this study. The data used to support the findings of this study are available from the corresponding author on request (contact J.C.D.L.F., josedelaflor81@yahoo.com or jflomer@mde.es). We confirm that all the figures and tables are the original work of this manuscript’s authors. All have been created by the authors of this manuscript, have not been adapted from other authors, and do not present an online link.
